# LATE EVALUATION OF DYSPHAGIA AFTER HELLER ESOPHAGEAL MYOTOMY WITH DOR FUNDOPLICATION FOR ACHALASIA

**DOI:** 10.1590/0102-6720201700030005

**Published:** 2017

**Authors:** Eduardo Rodrigues Zarco CÂMARA, Fernando Athayde Veloso MADUREIRA, Delta MADUREIRA, Renato Manganelli SALOMÃO, Antonio Carlos Ribeiro Garrido IGLESIAS

**Affiliations:** 1 General Surgery, Gaffrée and Guinle University Hospital, Federal University of the State of Rio de Janeiro,; 2 General Surgery Clinic, Fraga Filho University Hospital, Rio de Janeiro, RJ, Brazil

**Keywords:** Esophageal achalasia, Deglutition disorders, Fundoplication, Quality of life, Laparoscopy

## Abstract

**Background:**

All available treatments for achalasia are palliative and aimed to eliminate the flow resistance caused by a hypertensive lower esophageal sphincter.

**Aim:**

To analyze the positive and negative prognostic factors in the improvement of dysphagia and to evaluate quality of life in patients undergoing surgery to treat esophageal achalasia by comparing findings before, immediately after, and in long follow-up.

**Methods:**

A total of 84 patients who underwent surgery for achalasia between 2001 and 2014 were retrospectively studied. The evaluation protocol with dysphagia scores compared preoperative, immediate (up to three months) postoperative and late (over one year) postoperative scores to estimate quality of life.

**Results:**

The surgical procedure was Heller-Dor in 100% of cases, with 84 cases performed laparoscopically. The percent reduction in pre- and immediate postoperative lower esophageal sphincter pressurewas 60.35% in the success group and 32.49% in the failure group. Regarding the late postoperative period, the mean percent decrease was 60.15% in the success group and 31.4% in the failure group. The mean overall drop in dysphagia score between the pre- and immediate postoperative periods was 7.33 points, which represents a decrease of 81.17%.

**Conclusions:**

Reduction greater than 60% percent in lower esophageal sphincter pressurebetween the pre- and postoperative periods suggests that this metric is a predictor of good prognosis for surgical response. Surgical treatment was able to have a good affect in quality of life and drastically changed dysphagia over time.

## RESUMO

**Racional:**

Todos os tratamentos disponíveis para a acalásia são paliativos e visam eliminar a resistência ao fluxo causada por esfíncter esofágico inferior hipertenso.

**Objetivos:**

Avaliar os fatores prognósticos positivos e negativos na melhora da disfagia e avaliar a qualidade de vida em pacientes submetidos à cirurgia para tratar a acalásia esofágica, comparando os achados antes, imediatamente após e tardiamente à operação. Métodos : Foram estudados retrospectivamente 84 pacientes submetidos à operação de acalásia entre 2001 e 2014. O protocolo de avaliação com escores de disfagia comparou os escores pré-operatórios, do pós-operatório imediato (até três meses) e pontuação pós-operatória tardia (acima de um ano) para estimar a qualidade de vida.

**Resultados:**

O procedimento cirúrgico foi Heller-Dor em 100% dos casos, com 84 casos laparoscópicos. A redução percentual na pressão do esfíncter esofágico inferior pré e pós-operatório imediato foi de 60,35% no grupo de sucesso e de 32,49% no grupo de falha. Em relação ao período pós-operatório tardio, a redução percentual média foi de 60,15% no grupo de sucesso e 31,4% no grupo de falência. A queda média do escore de disfagia entre os períodos pré e pós-operatório imediato foi de 7,33 pontos, o que representa diminuição de 81,17%.

**Conclusões:**

A redução de mais de 60% da pressão do esfíncter esofágico inferior entre os períodos pré e pós-operatório sugere que ela é preditora de bom prognóstico cirúrgico. O tratamento cirúrgico foi capaz de contribuir na qualidade de vida e alterar em muito a disfagia em longo prazo.

## INTRODUCTION

Esophageal achalasia is an uncommon motility disorder of the esophagus, with an incidence of between 0.03 to 1 per 100,000 individuals^5^ with no preference in terms of age or gender^14^. This condition can be classified as idiopathic, chagasic, pseudoachalasia or linked to syndromic contexts^2^ ^,^ ^19^. It is secondary to an irreversible degeneration of Auerbach’s myenteric plexus of the esophagus, causing aperistalsis and the absence of lower esophageal sphincter relaxation^9^ ^,^ ^10^ ^,^ ^13^ ^,^ ^18^ ^,^ ^22^ ^,^ ^23^. Infection by *Trypanosoma cruzi* has had a major impact in South American countries, particularly in Brazil, and is known that approximately 5% of patients affected by Chagas disease develop achalasia^19^. This disease is insidious, and the main symptom is dysphagia. Patients are emaciated and have a poor quality of life (QoL), becoming incapacitated in terms of their labor activities^12^.

All available treatments for achalasia are palliative and are aimed at eliminating the flow resistance caused by a hypertensive lower esophageal sphincter. These treatments attempt to improve symptoms related to esophageal stasis, such as dysphagia and regurgitation^28^.

Treatment may be drug, surgical or endoscopically based^28^. Of the surgical procedures currently in use, surgical myotomy of the esophagogastric junction, with or without an anti-reflux valve, is considered to offer the best long-term outcomes^12^ based. In the last 20 years, this procedure has increasingly been performed using videolaparoscopy or robotic surgery. The advent of such techniques has decreased the morbidity associated with thoracotomy and myotomy by conventional surgery, which for years favored endoscopic pneumatic dilation as a first-line therapy^13^.

The aim of this study was to analyze positive and negative prognostic factors in the improvement of dysphagia and to evaluate QoL in patients undergoing surgery to treat esophageal achalasia by comparing findings before, immediately after, and in long postoperative follow-up.

## METHODS

This study was submitted to and approved by the Gaffrée and Guinle University Hospital Research Ethics Committee. Terms of Free and Informed Consent were presented and signed by patients when surgery was indicated.

Achalasia patients who underwent Heller esophagocardiomyotomy associated with partial Dor fundoplication by videolaparoscopy or Heller/Dor laparoscopy in the period from January 2001 to September 2014 were retrospectively studied. The surgical procedure was indicated in patients with achalasia who had megaesophagus grades I, II, III and selectively in cases of achalasia grade IV (Rezende classification^24^ ^,^ ^25^). Patients who underwent surgery in the esophagogastric junction, surgeries that were not performed by laparoscopy and the ones who could not attend follow-up were excluded from the study.

Patients were from the General Surgery Clinic, Clementino Fraga Filho University Hospital, Postgraduate Program in Surgery, PUC-Rio, and Gaffrée and Guinle University Hospital, Rio de Janeiro, RJ, Brazil. The evaluation protocol recorded the following characteristics: clinical history; physical examination; specific tests such as serology for Chagas disease; esophagus, stomach and duodenum seriography; upper digestive endoscopy and esophageal manometry before and later after surgery (pre-, immediate and late postoperative); dysphagia scores^26^, comparing dysphagia scores and QoL. Positive and negative prognostic factors that interfered to success or to failure of surgical treatment were also evaluated. Surgical treatment was considered to have failed when patients in the immediate or late postoperative period had persistent dysphagia, with a dysphagia score greater than or equal to 5, which were characterized as initial fail or late fail, respectively. Patients requiring complementary treatment such as pneumatic dilation, botulinum toxin injection or a new myotomy were also considered to be treatment fail.

The series was divided into two groups: Succ (success) and Fail (failure). To identify predictors of fail and positive prognostic factors, all demographics and characteristics of the cohort were tested, along with relevant aspects of surgery that could affect the overall result.

### Dysphagia evaluation

A dysphagia score was used to evaluate pre- and postoperative dysphagia^26^.The score evaluated the combination of the frequency and intensity of dysphagia using a points-based system described by Richards et al.^26^ (Table 1). The score totaled a maximum of ten points and was measured before and after surgery. The dysphagia scores were completed by checking the medical records and by interviewing patients at the clinic or by telephone. The last contact with patients was in 2014, allowing a later evaluation of the current degree of dysphagia compared with the pre- and immediate postoperative period.

**TABLE 1:** Dysphagia score^26^

Frequency of dysphagia 0-5 pts	pts	Severity of dysphagia 0-5 pts	pts	Total
Never	0	None	0	
Less than 1 day weekly	1	Very mild	1	
1 day weekly	2	Mild	2	
2 to 3 days weekly	3	Moderate	3	
4 to 6 days weekly	4	Moderately severe	4	
Daily	5	Severe	5	10

### Statistical analysis

For quantitative variables, the mean and/or median were calculated, and comparisons were made by applying Student’s t test to the mean and the Wilcoxon Rank Sum test to the median. The association between qualitative variables was performed using either the Chi-square or Fisher’s exact test. p value was <0.05. JMP software (SAS Institute) was used to perform statistical analysis.

## RESULTS

A total of 91 patients underwent surgery between January 2001 and September 2014. Seven who could not attend follow-up were excluded. Eighty-four undergoing HDL were, therefore, analyzed. The mean follow-up was 8.5 years and ranged from 1-13 years. Of these, 51 (60.7%) were female and 33 (39.3%) male. The mean age was 43.8 years (17-78). Dysphagia was the most common symptom, occurring in 100% of subjects, in varying intensities: to solids in 96%; to pasty foods in 64%; to liquids in 20%. Regurgitation of eaten food occurred in 33.3% of cases. There was reported weight loss in 78.18% with a mean weight loss of 10.3 kg (2-30 kg). Clinical malnutrition, that is, when body mass index was lower than 18.5, was reported in 8.5% of subjects.

A total of 16.07% of patients were classified as Rezende^24^ ^,^ ^25^ grade I, 57.1% as grade II, 21.4% as grade III, and 3.57% as grade IV, and this classification was used for megaesophagus of any etiology (idiopathic and chagasic). Upper digestive endoscopy showed normal esophageal mucosa in 62%, esophagitis in 13.95% and peptic ulcer disease (gastric or duodenal) in 27.9% of cases. Thirty underwent preoperative pneumatic dilation, representing 35.5% of the series, with a total of 35 pneumatic dilation with a mean of 1.66 dilations/patient (1-4 sessions).

The surgical procedure was Heller/Dor laparoscopy in 100% of cases, with 84 cases performed laparoscopically. There were no conversions. Mean surgery time was 147 min (90-260). The mean size of the esophagocardiomyotomy was 6 cm into the esophagus and 2 cm into the stomach, ranging from 5-12 cm total. Punctate perforation of the mucosa of the distal esophagus was identified in four patients during surgery. The mucosa was then sutured, covering it with gastric serosa for the Dor procedure. Ligation of the short vessels between the stomach and the spleen was performed in 14 (16.6%) patients. According to the Clavien-Dindo^7^ scale of complications, 5% were grade I and 2% grade II, while no cases were classified as grades III, IV, or V.

**FIGURE 1:**
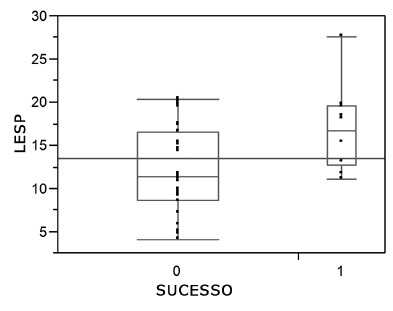
Mean LESP PO score: success and failure groups

**FIGURE 2:**
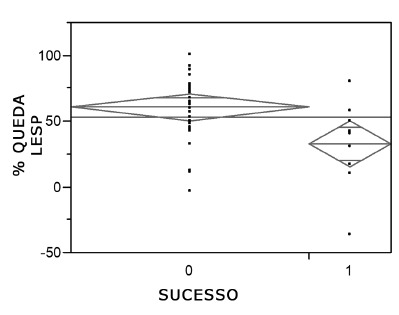
Mean percent drop in LESP: success and failure

The mean preoperative lower esophageal sphincter pressure (LESP) of the entire cohort was 33 mmHg (4.7-70), while the mean postoperative LESP was 13.3 mmHg (4.1-28.4, Table 2). The mean postoperative LESP was 11.36 mmHg for the Succ group and 16.75 mmHg for the Fail group. The p value was 0.022 (Figure 1). When the relationship between preoperative LESP and the absence of pneumatic dilation was analyzed; those who had one or more than one pneumatic dilations had different mean values: 0 pneumatic dilation=33 mmHg; 1 pneumatic dilation=28.39 mmHg; >1 pneumatic dilation=23.5 mmHg (p=0.043). The percent reduction in pre- and immediate postoperative LESP was 60.35% in the Succ group and 32.49% in the Fail group (Figure 2). Regarding the late postoperative period, the mean percent decrease was 60.15% in the Succ group and 31.4% in the Fail group (p=0.0087).

**TABLE 2:** Results of pre- and postoperative evaluations

Results	Preoperative	Early postoperative	Late postoperative
Mean LESP (mmHg)	33	-	13.3
Mean dysphagia score (0-10)	9.03	1.7	2.2
Group success: %LESP decrease	-	60.35	60.15
Group failure: %LESP decrease	-	32.49	31.4
% Success	-	89.3	84.5
% Failure	-	10.7	15.5

LESP=lower esophageal sphincter pressure

**FIGURE 3:**
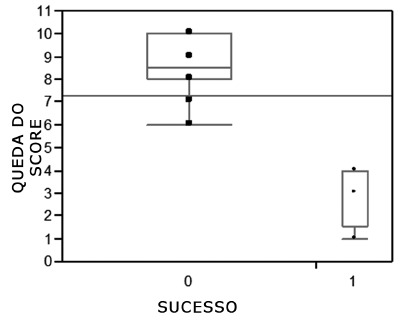
Plot of the pre- and postoperative decrease in dysphagia score vs. success (0) and failure (1)

The mean preoperative scores for dysphagia frequency and intensity were 4.7 and 4.1 points, respectively. The total mean preoperative score was 9.03 points (maximum 10 points). When the dysphagia score was applied in the immediate postoperative period, the mean dysphagia frequency score was 0.9 points, the mean dysphagia intensity was 0.8 points, and the mean overall score was 1.7 points. The mean overall drop in dysphagia score between the pre- and immediate postoperative periods was 7.33 points, which represents a decrease of 81.17% (Figure 3, p=0.0001). In relation to the late postoperative period, the overall mean was 2.2 points, with a late postoperative fall of 6.83 points, representing 75.6% (p=0.03, Table 2). There was no significant difference between pre- and immediate postoperative periods and late postoperative fall.

The improvement in QoL was evaluated in terms of initial and late Succ, using a drop in the dysphagia score to less than 5 in the comparison between the preoperative and immediate and late postoperative periods. Initial Succ was evident in 75 of 84 patients, representing 89.3% of the series, while nine, or 10.7% of the series, were considered to represent initial Fail of the surgical treatment. In relation to late Succ, 71 patients, or 84.5%, were considered to be Succ, while 13, or 15.5% (four patients more than those considered initial Fail), were deemed late Fail (p=0.03, Table 2).

The Succ/Fail groups did not differ in any aspect regarding studied variables.

## DISCUSSION

The study was designed as retrospective with prospective data collection. Data were obtained from medical records, direct interviews and telephone contact. These data were used to analyze the long-term QoL of patients undergoing achalasia surgery, along with Succ or Fail predictors. There is doubt in the literature regarding the efficacy of HDL in controlling this disease over several years^16^. Some published papers have reported excellent or good results ranging from 78-93%, with no single criterion for Succ or Fail in surgical treatment of achalasia^6^ ^,^ ^4^ ^,^ ^16^ ^,^ ^17^ ^,^ ^29^. The follow-up was performed to evaluate patients’ long-term responses.

One of the predictors of Succ or adequate surgery response that has already been noted in the literature is a preoperative LESP of >35 mmHg. The chance of Succ is 21 times higher in individuals with a high LESP score (>35 mmHg) than in LESP <35 mmHg^21^ ^,^ ^24^. The behavior of LESP in this series follows the trends seen in other studies, with a mean LESP value of 33 mmHg preoperatively and 13.3 mmHg postoperatively (p<0.05). We can presume that prior Heller cardiomyotomy was able to decrease LESP effectively, as the population before and after surgery behaved differently and antagonistically with regard to this variable. According to our results, comparing the reduction in LESP, both between the pre- and immediate postoperative period and between the pre- and late postoperative period, revealed a percent decrease that was greater than 60% (p<0.001). This result represents a good early and late prognostic factor. Based on the postoperative LESP result alone, with the surgical goal of returning the LESP to near normal, only 7.1% of cases continued to have LESP above 20 mmHg^16^. The majority of subjects, 92.9%, displayed a decrease in the LESP to near normal values.

Another variable analyzed was pneumatic dilation. This procedure was performed in 30 patients, totaling 35 dilations. When the relationship between preoperative LESP and patients who did not have pneumatic dilation, one pneumatic dilation and more than one was analyzed statistically; the means were different as previously described (p=0.043). We presume that the pneumatic dilation was able to modify these three populations in relation to their preoperative pressure values. The relationship between having had pneumatic dilation preoperatively and the postoperative LESP result was also studied. The mean postoperative LESP in those who did not have pneumatic dilation was 15.26 mmHg and was 10.20 mmHg in those who did have it (p=0.0045), i.e., preoperative pneumatic dilation significantly affected the final postoperative LESP by lowering it, contrary to expectations. This finding may be related to the fact that previous pneumatic dilation also lowered preoperative LESP. These patients would therefore already have a lower one before surgery, which may have been reflected in the measurements taken after surgery. Another fact that supports this reasoning is that we did not find a significant difference (p=0.47) in the relationship between prior pneumatic dilation and the percent reduction in LESP. This variable is calculated from pre- and postoperative LESP values and therefore negate the influence of pneumatic dilation. However, having had prior pneumatic dilation, regardless of the number of times, did not significantly affect Succ or Fail (p=0.35), nor did it increase the chance of surgical complications or morbidity. Most published studies also do not show this correlation^11^ ^,^ ^27^. Smith et al.^27^ observed an adverse outcome in their series of 209 patients, with 74% having undergone prior endoscopic therapy. Their study showed a higher surgical complication rate, with mucosal opening in 17, of whom 15 had undergone prior endoscopic treatment (pneumatic dilation or Botox). These researchers also reported having encountered fibrosis and difficulty in dissecting the hiatus and perimediastinal region. Another report described significantly increased HDL surgical times in patients who had previously undergone pneumatic dilation or botulinum toxin injection^11^.

The Fail criteria were adopted based on the dysphagia score, with patients who had persistent dysphagia one year postoperatively, with a dysphagia score of greater than or equal to 5, as well as those who required complementary treatment such as pneumatic dilation or botulinum toxin injection, considered as surgical treatment fail. None of the 22 patients classified as Fail required reoperation and were managed with pneumatic dilation or a change of diet. The percentage decrease in the dysphagia score was 81.17% in the immediate postoperative period (p<0.0001). In the late postoperative period, the drop was 75.6% (p=0.03). We believe that these data, sustained over a mean follow-up of 8.5 years, suggests that the surgical treatment had a large impact on QoL, drastically changing the main symptom of dysphagia in the long term.

The increase in QoL was evaluated by the initial and late Succ demonstrated by a reduction in the dysphagia score to less than 5 from the preoperative to the immediate and late postoperative periods. Initial Succ was evident in 75 of the 84 patients undergoing surgery, which represents 89.3% of the series, while nine, or 10.7%, were considered to represent initial fail of the surgical treatment. In relation to late Succ, 71 patients, or 84.5%, were considered Succ, while 13, or 15.5%, were late Fail (p=0.03). The postoperative follow-up period revealed a clear favorable prognosis and did not affect treatment fail. This finding was not mentioned in various prior studies and may be considered unprecedented. Confirmation of these data should be expected in subsequent studies.

When considering all studies analyzed evaluating Heller esophagocardiomyotomy associated with prior Dor fundoplication and its variants, it can be concluded that HDL is safe surgery, with a short hospitalization time and a high success rate in short and long term^3^ ^,^ ^21^. These procedures have a significant effect on the main symptom, dysphagia, in addition to having low morbidity and mortality. Such surgery is therefore recommended in the management of achalasia^1^ ^,^ ^3^ ^,^ ^8^ ^,^ ^15^ ^,^ ^20^.

## CONCLUSION

Reduction greater than 60% in LESP between the pre- and postoperative periods suggests that this metric is a predictor of a good prognosis for surgical response. Surgical treatment was able to have a significant effect in QoL and drastically changed dysphagia in long follow-up.
